# Gametocyte Sex Ratio: The Key to Understanding *Plasmodium falciparum* Transmission?

**DOI:** 10.1016/j.pt.2018.12.001

**Published:** 2019-03

**Authors:** Fitsum G. Tadesse, Lisette Meerstein-Kessel, Bronner P. Gonçalves, Chris Drakeley, Lisa Ranford-Cartwright, Teun Bousema

**Affiliations:** 1 Radboud Institute for Health Sciences, Radboud University Medical Centre, Nijmegen, The Netherlands; 2 Armauer Hansen Research Institute (AHRI), Addis Ababa, Ethiopia; 3 Institute of Biotechnology, Addis Ababa University, Addis Ababa, Ethiopia; 4 Department of Immunology and Infection, London School of Hygiene and Tropical Medicine, London, UK; 5 Institute of Biodiversity, Animal Health and Comparative Medicine, College of Medical, Veterinary and Life Sciences, University of Glasgow, Glasgow, UK; 6 These authors contributed equally

**Keywords:** gametocytes, sex ratio, transmission, *Plasmodium falciparum*, diagnosis, commitment to gametocytes

## Abstract

A mosquito needs to ingest at least one male and one female gametocyte to become infected with malaria. The sex of *Plasmodium falciparum* gametocytes can be determined microscopically but recent transcriptomics studies paved the way for the development of molecular methods that allow sex-ratio assessments at much lower gametocyte densities. These sex-specific gametocyte diagnostics were recently used to examine gametocyte dynamics in controlled and natural infections as well as the impact of different antimalarial drugs. It is currently unclear to what extent sex-specific gametocyte diagnostics obviate the need for mosquito feeding assays to formally assess transmission potential. Here, we review recent and historic assessments of gametocyte sex ratio in relation to host and parasite characteristics, treatment, and transmission potential.

## Highlights

Recent RNA sequencing studies have uncovered a number of *P. falciparum* gametocyte sex-specific targets and provided new insights in gametocyte biology.

After decades when gametocyte sex-ratio research was restricted to nonhuman malarias or *in vitro* experiments, molecular tools for assessing gametocyte sex ratio are now increasingly available for use in natural *P. falciparum* infections.

Evidence that gametocyte sex ratio is influenced by total gametocyte density and antimalarial treatment, and improves predictions of transmission potential, highlight the relevance of understanding the gametocyte sex ratio during natural infections.

The finding that the most widely used *P. falciparum* gametocyte marker *Pfs25* is expressed predominantly by female gametocytes and has non-negligible levels of background expression in asexual parasites necessitates a re-evaluation of existing gametocyte data.

## Sexual Commitment and First Appearance of Male and Female *P. falciparum* Gametocytes

The transmission of malaria from human to mosquito depends on the presence of **gametocytes** (see Glossary), sexual-stage *Plasmodium* parasites, in the peripheral blood. The development of gametocytes of *P. falciparum* takes 8–12 days and it involves transitions from committed rings, via early and intermediate stage gametocytes that are sequestered in the bone marrow and spleen (stages I–IV) 1, 2, 3, to mature male and female stage V gametocytes [4]. In other *Plasmodium* species, such as *P. vivax, P. chabaudi, P. vinckei*, and *P. gallinaceum*, gametocytes develop much more rapidly, typically within 3 days [5]. In **controlled human malaria infections** (CHMIs) less than 10% of all asexual *P. falciparum* parasites commit to form gametocytes 6, 7, and mature gametocytes typically comprise less than 5% of the circulating parasite biomass in natural infections [8]. **Sexual commitment** happens before the stage of **schizogony** 9, 10, 11, 12, and all merozoites derived from one schizont develop into either microgametocytes (males) or macrogametocytes (females) 13, 14. The lack of sex chromosomes in haploid *Plasmodium* hampers our understanding of the commitment to sexual differentiation and the timing of **sex determination** [15]. Sex determination could occur at the same moment when commitment to sexual differentiation is determined [13], or alternatively via a two-stage process in which sex determination happens after the decision on commitment to sexual differentiation. At the molecular level, the nuclear protein *P. falciparum* gametocyte development 1 (GDV1) triggers the first known part of the molecular cascade of commitment and acts as the upstream regulator of the DNA-binding protein AP2-G (PF3D7_1222600), by antagonizing heterochromatin protein 1 repression of AP2-G transcription [16]. Sufficient activation of AP2-G, the master transcriptional regulator of gametocytogenesis, represents the ‘point of no return’ in commitment to both male and female gametocytes 11, 17, 18. The molecular basis of sex determination, and its timing during development, is currently unknown.

Here, we summarize historic and recent estimates of gametocyte **sex ratio** in natural infections and the limitations of older estimates that relied on microscopy. We present the merits and limitations of molecular tools for quantifying gametocyte sex ratio and review evidence for a sex-specific effect of antimalarial drugs on circulating gametocytes and the implications for malaria transmission potential.

## Microscopy Is an Imperfect Tool for Quantifying Gametocyte Sex Ratio

**Figure 1:**
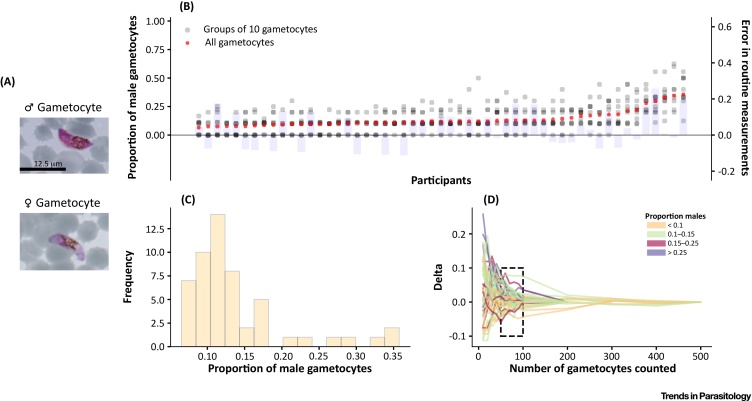
Intensive Microscopy-Based Quantification of Gametocyte Sex Ratio. In this figure, we use data generated by one of the authors (C.D.) in 1992–1993 to illustrate the likely error in gametocyte sex ratio estimation when determining the sex of only a few gametocytes per sample as routinely done in epidemiological studies. Briefly, 100–500 gametocytes were sexed per blood smear in 53 samples from 43 individuals living in malaria-endemic regions in The Gambia, and sex-specific counts were recorded in groups of 10 gametocytes. Light microscopy images of male and female gametocytes stained with Giemsa’s stain are shown in (A) for illustration. Female gametocytes are blue/violet (as opposed to pink males), more crescent-shaped, and have more compact nuclei and more centrally located pigment. In (B), *x* coordinates represent data from different samples, partially transparent to allow visualization of overlapping data points: red crosses (left *y* axis) correspond to the proportions of gametocytes identified as male-based on data from groups of 10 gametocytes or the total number of gametocytes, respectively; light blue bars represent the difference (right *y* axis) between the proportion of male gametocytes when considering only the first 10 gametocytes observed in each smear versus the proportion calculated based on the total number of gametocytes. This difference exemplifies the error that might occur in routine measurements that quantify only a limited number of gametocytes. In (C), the distribution of the proportion of male gametocytes in the different thick smears analyzed, based on the total number of gametocytes, is shown. Panel (D) presents the progressive reduction in error as the number of gametocytes counted increases. In this panel, the *x* axis corresponds to the cumulative number of gametocytes sexed, and the *y* axis corresponds to the difference in the proportion of male gametocytes relative to the same proportion when estimated based on all gametocytes observed in the smear. Each line represents a different sample, and colors relate to the overall proportion of male gametocytes in the smear. The rectangle delimited by the dashed lines encloses error values between −0.1 and 0.1 when 50–100 gametocytes were counted. Only data from thick smears were used in this figure.

In natural infections of *P. falciparum*, the sex ratio of circulating gametocytes is mostly female biased [19]. Only a few epidemiological studies have quantified female and male gametocytes separately with sex ratios ranging from a mean of ∼three to five females to one male (Table 1) 20, 21, 22, 23. Previously reported sex ratios are based mainly on microscopic examination of Giemsa-stained thick or thin blood smears, relying on subtle morphological differences between mature (stage V) macrogametocytes and microgametocytes [24] (Figure 1). Gametocyte sex determination by microscopy is challenging due to the typically low gametocyte densities and the difficulty of allocating a sex to the sparsely observed gametocytes 22, 25, 26, 27, 28. As an illustration of their sparseness, in a recent detailed study on gametocyte carriage in Burkina Faso, only 20% (95% confidence interval [CI] 14–27%) of patent asexual parasite carriers had microscopically detected gametocytes, and only 7% (95% CI 4–12%) had more than two gametocytes observed whilst enumerating against a conventional number of 500 white blood cells (∼0.06 μl of blood microscopically screened) [29]. The implications of low observed gametocyte counts for sex ratio precision are presented in Figure 1, indicating that observations based on 500 white blood cells can rarely lead to accurate gametocyte sex ratio estimates. Only by observing 50–100 gametocytes can a reproducible sex ratio be calculated (Figure 1D) 8, 19, and this is hardly ever attainable by routine microscopy. Microscopic investigation of gametocyte sex can be improved by concentrating gametocytes with magnetic enrichment [30], assuming it enriches male and female gametocytes equally, allowing more gametocytes to be examined. The accuracy of differentiating male and female gametocytes can be further improved by targeting proteins or transcripts that are preferentially expressed in a specific sex using immunofluorescence or probe-based hybridization assays, respectively. Assays that target stage-specific expression of transcripts, such as *Pf77* (PF3D7_0621400) and *Pfg377* (PF3D7_1250100), both enriched in female gametocytes 31, 32 and *Pfg27* (PF3D7_1302100, detecting both sexes) 33, 34 have been reported previously. Immunofluorescence assays based on antibodies that bind proteins specific for early gametocytes such as Pfs16 (PF3D7_0406200) 14, 35, male gametocytes (α-tubulin II, PF3D7_0422300) 13, 14, 36, or female gametocytes (Pfg377, PF3D7_1250100) [13] have also been used. Insights into sex differences in the transcriptional and proteomic makeup are summarized in Box 1. These immunofluorescent assays alleviate common problems that complicate microscopic examination but their reliance on fluorescence microscopy greatly affects their deployment in the field. The recent development of molecular assays to quantify male and female gametocytes may allow more robust sex ratio determination at low gametocyte densities and easier application in field settings.

**Table 1:** Summary of Studies That Evaluated Sex Ratio in Natural Infections

Setting	Population	Proportion male^a^	Tool	Refs
Mali, Burkina Faso, Cameroon	Asymptomatic gametocyte carriers	0.14–0.51	qRT-PCR	[23]
The Netherlands	Controlled human malaria infection volunteers	0.29	qRT-PCR	[7]
Mali	Asymptomatic gametocyte carriers	0.30	qRT-PCR	[62]
Australia	Controlled human malaria infection volunteers	0.20	qRT-PCR	[6]
Kenya, Mali	Asymptomatic gametocyte carriers	0.36	qRT-PCR	[34]
Nigeria	Symptomatic malaria patients	0.21	Microscopy	[75]
Nigeria	Symptomatic children	0.34	Microscopy	[87]
Nigeria	Symptomatic malaria patients	0.22	Microscopy	[88]
Nigeria	Symptomatic malaria patients	0.75	Microscopy	[76]
India	Symptomatic malaria patients	0.31	Microscopy	[89]
Nigeria	Symptomatic malaria patients	0.20	Microscopy	[78]
Nigeria	Symptomatic malaria patients	0.05	Microscopy	[90]
Nigeria	Symptomatic malaria patients	0.14	Microscopy	[91]
Senegal	Symptomatic children	0.15	Microscopy	[92]
Senegal	Total population	0.35	Microscopy	[93]
Nigeria	Asymptomatic children	0.42	Microscopy	[77]
Tanzania	Not applicable^b^	0.34	Indirect: calculated from inbreeding coefficient	[94]
The Gambia	Not applicable^c^	0.22	Indirect: calculated from inbreeding coefficient	[94]
Sudan	Not applicable^d^	0.07	Indirect: calculated from inbreeding coefficient	[94]
Cameroon	Symptomatic malaria patients	0.22	Microscopy	[22]
Papua New Guinea	Not applicable^b^	0.04	Indirect: calculated from inbreeding coefficient	[95]
Papua New Guinea	Not indicated	0.18	Microscopy	[96]

a Proportion male is defined as the proportion of all gametocytes that is male, [male gametocytes/(male + female gametocytes)].

b Inbreeding coefficients calculated directly from oocyst selfing rates from mosquitoes.

c Inbreeding coefficients calculated from allele frequencies in blood-stage infections, but sex ratios not determined in these patients. Patients were symptomatic children.

d Inbreeding coefficients calculated from allele frequencies in blood-stage infections, but sex ratios not determined in these patients. Patients were symptomatic children and adults.

Box 1 **The Development of Male and Female Gametocytes**

Molecular mechanisms underlying the differentiation switch towards becoming male or female gametocytes remain largely unknown. Candidate genes that may be associated with sex-specific differentiation include the *AP2-G2* in *P. berghei* [18], the *Puf* family of translational repressors in *P. falciparum* 97, 98, and MAPK1 and MAPK2 as potential regulators of female and male gametocytogenesis in *P. falciparum*, respectively [63].

Male gametocytes are terminally differentiated forms with one last task ahead; producing motile microgametes upon activation. **Proteomic** and **transcriptomic** data reflect this, showing upregulation of genes involved in nucleic acid metabolism, DNA replication, and axoneme formation. Female gametocytes, on the other hand, contain many proteins and transcripts intended for longer term sustainment of postfertilization stages, including genes involved in protein biosynthesis, degradation, transport, and metabolic activity. Another important female gametocyte-specific mechanism is **translational repression** in which stored transcripts support cellular processes immediately after fertilization, when transcription is absent for multiple hours 99, 100. The deletion of genes involved in the maintenance of translationally repressed mRNA transcripts, such as DOZI [101] and CITH [102] in *P. berghei*, results in full developmental arrest during ookinete formation, although fertilization is successful. The first indication that translational repression is also active in *P. falciparum* was obtained through deletion of *Puf2*, which resulted in altered expression of genes known to be translationally repressed, such as *Pfs25* and *Pfs28* 103, 104. More recent proteomics and transcriptomics data have uncovered a large-scale translational repression of hundreds of transcripts in *P. falciparum* female gametocytes [41].

Alt-text: Box 1

## New Molecular Techniques Improve Sex Ratio Quantification

Gametocyte detection has been improved in the last decade with the introduction and wider use of molecular detection tools. Baker *et al.* reported the first molecular assay for an indirect detection of gametocyte sexes based on *in situ* hybridization using a *Pf77* RNA probe that targeted female gametocytes [31]. Assays were subsequently developed targeting the *Pfs25* (PF3D7_1031000) transcript, which was considered highly specific for mature gametocytes, using qRT-PCR 37, 38 or real-time quantitative nucleic acid sequence-based amplification (QT-NASBA) [39]. Whilst highly sensitive, with an estimated sensitivity around 0.1 gametocytes per μl in 50–100 μl blood samples, this marker (*Pfs25*) has recently been shown to be expressed predominantly by female gametocytes 40, 41. Drew and Reece reported the first molecular approach to gametocyte sex ratio determination [28] in the rodent malaria parasite *Plasmodium chabaudi* using targets that were previously described in a **proteomics** study in *Plasmodium berghei* [42], based on the quantification of total gametocytes by common gametocyte gene 1 (*CG1* or *PSOP1*: PCHAS_0620900) RNA, and male gametocytes by the male gametocyte-specific gene 1 (MG1, a putative dynein heavy chain: PCHAS_0417000). Schneider and colleagues [40] reported the first qRT-PCR assays that quantify female and male gametocytes in *P. falciparum* through quantification of a combination of female-enriched (*Pfs25*) and male-enriched (*Pfs230p*) transcripts. The higher limit of detection for male (1.8 male gametocytes/μl) compared to female gametocytes (0.3 female gametocytes/μl) in this assay, and the typical female bias in natural infections, may affect the sensitivity of sex ratio determination at low gametocyte densities. Recently, additional male gametocyte-enriched transcripts (*Pf13*, PF3D7_1311100 and *PfMGET*, PF3D7_1469900) were identified that improved the detection limit of male gametocytes to the level comparable with female gametocytes 34, 43. Details of all currently used transcripts for quantifying male and female gametocytes are provided in Box 2. Of note, none of the gametocyte markers are exclusively expressed in gametocytes of one sex 34, 43, and low levels of ‘gametocyte transcripts’ are also detectable in asexual-stage parasites [44]. The latter has implications for earlier statements on the high prevalence of gametocytes in *P. falciparum*-infected individuals. Whilst it remains plausible that (nearly) all infections produce gametocytes 6, 7, the initial wave of high asexual parasitemia that is observed in clinical malaria cases may coincide with false-positive gametocyte signals. This may result in an overestimation of gametocyte prevalence at the moment of sampling, in particular in clinical malaria cases [19].

Box 2 **Marker Transcripts for Molecular Detection of Male and Female Gametocytes**

Mature male and female gametocytes can be distinguished on the basis of light microscopy on Giemsa-stained thin smears [24], by electron microscopy 10, 105, by an immunofluorescence assay based on female- or male-enriched proteins and monoclonal antibodies 14, 35, and by sex-enriched transcripts 31, 32. Sex is not determined chromosomally, since haploid clone lines from a single haploid parasite can generate both male and female gametocytes 16, 106. Gametocyte identification is thus solely based on quantification of stage-specific expression of transcripts.

Suitable targets for gametocyte diagnostics require high and stable expression of the gene in the stage and sex of interest. In Table I, the characteristics of the most important targets for gametocyte detection and quantification are summarized. Putative gametocyte sex markers rely on abundant transcript expression at the gametocyte stage and not in the asexual blood stage, with expression at other life stages being irrelevant. Importantly, *Pfs25* expression in asexual blood-stage parasites is not completely absent 43, 44, 107 but approximately 100 000-fold reduced. This estimate is similar for other reported markers [44], and hence low gametocyte transcript numbers may be derived from asexual parasites. Gametocyte prevalence thus needs to be interpreted with caution, especially at higher parasite burdens (above 1000 parasites/μl). The high rate of expression of some male markers (*Pf13* and *Pf230p*) [43] in mature trophozoites raises questions on the utility of these transcripts as differential sex-specific markers. During the dynamics of sequestration in the bone marrow and release into the peripheral blood, transcripts derived from these stages may be detected and could affect sex-specific quantification.

Differential *P. falciparum* [41] and *P. berghei* [108] gene expression studies per sex are resources for identification of potential candidates and may be particularly informative when integrated with asexual expression levels [109]. These studies have uncovered an increasing number of ‘sex-specific transcripts’ (Table I) that are, however, not sex-specific in the strict sense but rather sex-enriched. The commonly used (female) gametocyte marker *Pfs25* shows lower expression in male gametocytes compared to females, ranging from 35-fold lower as detected by RNAseq [41] to 200-fold lower using qRT-PCR [44]. Of note, spurious transcript expression in asexual parasites cannot be linked to (functional) protein expression, and so the biological relevance of transcripts of ‘gametocyte genes’ in asexual parasites remains unknown. For any mRNA target, qRT-PCR specificity can be enhanced if an intron-spanning region is targeted, as this ensures amplification of DNA derived from transcripts only, rather than genomic DNA, and avoids the need for DNase treatment that may decrease sensitivity of the assay through RNA damage [110]. The reliability of sex ratio determination can be further enhanced by multiplexing targets with the above features in one assay. For example, a multiplexed qRT-PCR that targets female gametocyte-specific (PF3D7_0630000) and total parasite (18S rRNA) transcripts, with no DNase treatment step, was introduced recently [111]. Similarly, a multiplex assay for male and female gametocytes with intron-spanning primers for both male (*PfMGET*) and female (*CCp4*) targets was recently proposed as a more scalable and robust approach to molecular gametocyte sex ratio assessments.

Alt-text: Box 2

**Table I:** Gametocyte- and Sex-Specific Transcripts Currently Used in Molecular Detection Methods

Gene ID name	Limit of detection	Description/putative function	FG:MG ratio^a^	Remarks	Refs
PF3D7_1031000 Pfs25	0.01 FG/μl	Zygote and ookinete surface protein, necessary for infectivity, transmission-blocking vaccine candidate	35.58		37, 112
PF3D7_0630000 No alias	0.3 *gametocytes*/μl	CPW-WPC family protein	47.83	Contains nine introns	[111]
PF3D7_1351600 PfGK	0.3 FG/μl	Glycerol kinase	41.41		[43]
PF3D7_0903800 CCp4	0.1 FG/μl	LCCL domain-containing protein, sexual-stage adhesion	38.49	Contains one intron	[44]
PF3D7_0208900 Pfs230p	1.8 MG/μl	Gamete surface protein, knock out has no effect on fertilization	0.02		[40]
PF3D7_1469900 PfMGET	0.01 MG/μl	Conserved *Plasmodium* protein, unknown function	0.02	Contains two introns	[34]
PF3D7_1311100 Pf13	22 MG/μl	Meiosis-specific nuclear structural protein	0.005		[43]
PF3D7_1319800 Pfg17	0.1 *gametocytes*/μl	Conserved *Plasmodium* protein, unknown function	0.60	Total gametocyte marker	[113]

a Ratio of transcript expression as detected by RNAseq in sex-sorted gametocytes, expressed as FPKM (fragments per kilobase million) values in female over male gametocytes [41].

## Sex Ratio Plasticity in Response to a Changing Environment

Recently, molecular methods to accurately sex gametocytes have been used to assess the first appearance of mature male and female gametocytes after experimental infections. Gametocyte appearance is estimated to occur 10 days post blood-stage inoculum [6] or 8.5–12 days after the first detection of asexual parasites in the peripheral blood [7], supporting previous suggestions of gametocyte commitment during the first erythrocytic cycle of asexual parasites 45, 46. During infections, malaria parasites may respond to environmental cues to alter their investment into the transmission stages (reviewed in [19]). In addition to an overall increased investment in gametocytes 47, 48, malaria parasites may adjust their sex ratio to maximize the transmission success when under stress, such as that induced by immunity developing as the infection progresses. In an early observation of an individual deliberately infected with *P. falciparum*, the sex ratio changed from female biased to equal sexes over 13 days [49].

Data from the avian parasite *Plasmodium gallinaceum* and rodent malaria *Plasmodium vinckei* further suggest that the sex ratio in *Plasmodium* infections varies substantially during the course of an infection 50, 51, possibly in response to the host’s immune response, anemia, and gametocyte density 50, 52, 53. A change in concentration of erythropoietin, a hormone that controls erythrocyte production, causes an increase in the **proportion of male gametocytes** in *P. vinckei* and increases overall gametocyte density in *P. gallinaceum* [54] and *P. chabaudi* [55].

Competition between parasites of different genotype can also affect sex ratio. Hamilton’s theory of ‘local mate competition’ (LMC) [56] states that female-biased sex ratios are optimal when genetically-related males compete for mates; the optimal sex ratio for malaria parasites depends on the rate of self-fertilization (inbreeding rate), where sex ratio (here: proportion of male gametocytes) = (1 − *f*)/2, and *f* is Wright’s inbreeding coefficient [57]. When individuals are infected with a single or a low number of distinct parasite genotypes, inbreeding will be high and a female-biased sex ratio is predicted. As inbreeding levels fall with increased diversity of parasite genotypes within the infection, the optimal sex ratio will approach 0.5. In an experimental test of this theory, using the rodent malaria *P. chabaudi* where genetic diversity was adjusted deliberately, sex ratio became more male-biased as the number of genotypes present increased [52].

The effect of sex ratio on transmission success may depend on total gametocyte density [23]. In low-density infections, a larger investment in male gametocytes is favorable to increase the chance that all females are fertilized 58, 59. As male gametocytes can produce up to eight microgametes upon activation, female-biased gametocyte sex ratios contribute to a more balanced number of macro- and microgametes in the mosquito midgut [51]. At high gametocyte densities, a male-biased sex ratio leads to less efficient transmission [60], favoring a less male-biased ratio. Recently, molecular gametocyte sex ratio assays supported the density-dependency of sex ratio with a higher proportion of male gametocytes in low-density infections [23]. The same study also suggested that quantifying male and female gametocytes allowed a better prediction of mosquito infection rates as compared to previous estimates that quantified female gametocytes only [61], and that the number of male gametocytes may become a limiting factor in determining transmission success at low densities. These findings require replication, but potentially have important implications for gametocyte diagnostics. If male gametocytes are indeed a limiting factor for transmission, diagnostics that quantify male gametocytes might be better indicators of transmission potential than female gametocyte diagnostics that are currently most widely used.

Differences in the longevity of male and female gametocytes can also contribute to sex ratio differences. Two studies have also hinted towards a shorter circulation time of male gametocytes upon clearance of the asexual progenitors 7, 62, although a third study observed identical circulation times [62]. An early study on induced *P. vivax* malaria also reported a shorter lifespan of male *P. vivax* gametocytes [49]. The different circulation time estimates may be influenced by differences in the ability to detect male and female gametocytes (including a lower sensitivity of male markers 6, 34) but are potentially highly important to understand the duration of infectiousness of gametocyte carriers. There is currently no published evidence on differences between male and female gametocytes in maturation or longevity *in vitro*. Longitudinal studies are needed to understand the dynamics of male and female gametocyte densities during natural infections and their impact on transmissibility.

If *Plasmodium* can indeed alter sex ratio in response to environmental cues, an essential outstanding question is what governs sex allocation. Candidate genes that may be associated with sex-specific regulation (other than or in addition to AP2-G) include MAPK1 (PF3D7_1431500) as potential regulator of female gametocytogenesis, and MAPK2 (PF3D7_1113900) as possible regulator of male gametocytogenesis [63]. Given the 10–12 day maturation time in *P. falciparum* infections and early commitment to the sexual stage (and plausibly gametocyte sex), any response to environmental triggers must involve significant ‘planning ahead' and cross-talk between potentially very low numbers of gametocytes or precursors. Even if environmental signals are as rapidly translated into elevated gametocyte conversion rates as recently shown for *P. chabaudi* [64], it will only have an effect after full maturation. One speculative option that would allow *P. falciparum* to rapidly respond to environmental triggers is the bone marrow reservoir for gametocytes. It is conceivable that mature gametocytes may not all be immediately released upon completion of maturation but may be in part retained for release upon environmental stimuli.

Our understanding of environmental factors that influence gametocyte sex ratio is incomplete. Moreover, some of the ‘known’ stimuli for overall investment in gametocyte production appear uncertain when assessed with current methodologies [65] and thus require re-examination. Useful markers to examine stimuli for gametocyte production and sex allocation include parasite AP2*-*G transcripts 11, 17, 18, the host factor lysophosphatidylcholine [65] and molecular markers to distinguish early gametocytes (GEXP5, PF3D7_0936600 66, 67), immature gametocytes (*Pfs16*, *PF14_0748*) [35], and mature gametocytes of different sexes (Table 1) 68, 69, 70. Potential stimuli include anemia, antimalarial treatment, host immunity, and coinfections with other *Plasmodium* parasites or clones [19].

## Antimalarials Influence Gametocyte Sex Ratio

**Figure 2:**
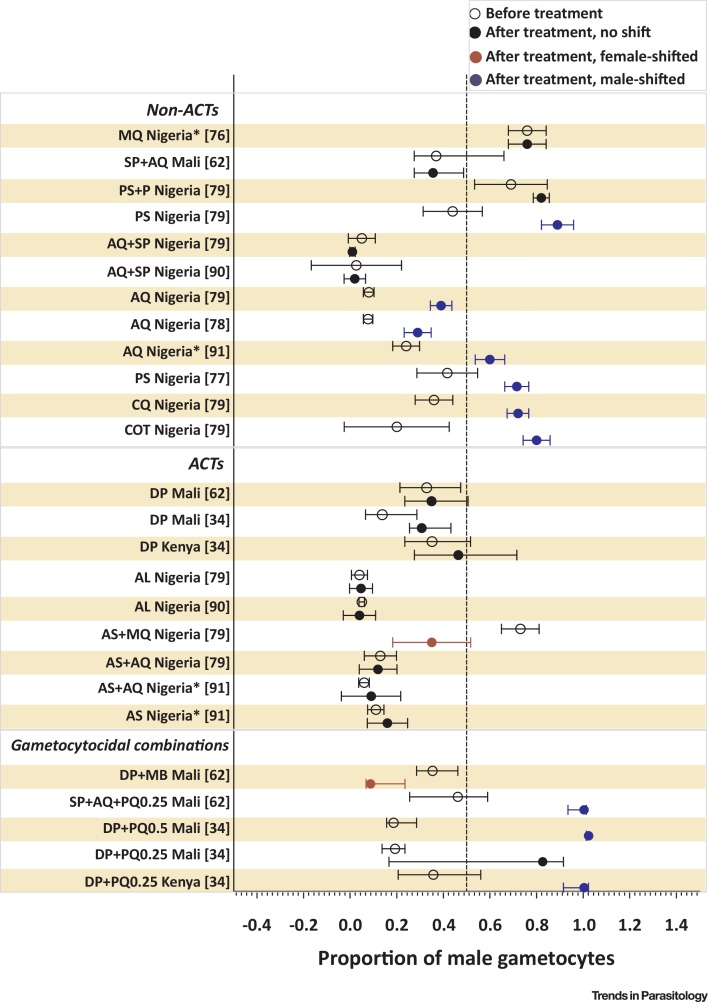
Forest Plot on Proportion of Male Gametocytes before and after Treatment. This plot summarizes available clinical trial data on the effect of antimalarials on gametocyte sex ratio. Indicated are the proportion of gametocytes that were male before treatment (open symbols) and after treatment (closed symbols; at day 7 post-treatment – if earlier, indicated with asterisks). Study drugs are indicated on the y axis and grouped by nonartemisinin-based combination therapies (non-ACTs), ACTs, and drug combinations with a gametocytocidal compound. Blue symbols indicate a shift towards males, and red indicates a shift towards more females surviving; based on nonoverlapping confidence intervals. In most cases estimates indicate the mean and 95% confidence interval; for two studies 34, 62 it is median and interquartile range. Abbreviations: ACT, artemisinin-combination treatment; AL, artemether-lumefantrine; AQ, amodiaquine; AS, artesunate; COT, cotrimoxazole; CQ, chloroquine; DP, dihydroartemisinin-piperaquine; MQ, mefloquine; P, probenecid; PQ, primaquine; PS, Pyrimethamine–sulfadoxine; SP, sulfalene–pyrimethamine. See also 76, 77, 78, 79, 90, 91.

Malaria parasites may alter their investment in asexual replication and transmission in response to drug pressure. Treatment with subcurative doses of antimalarial drugs has been shown to increase the rate of gametocytogenesis in the rodent malaria parasite *P. chabaudi* [71] and in laboratory strains of *P. falciparum* [72]. *P. falciparum* parasites may respond to their proliferation state rather than directly to the presence of drugs. Drug pressure may thus lead to an initial increased investment in asexual proliferation (aimed towards survival within the infected host) until survival is unlikely and a terminal investment in transmission occurs 64, 73. A direct effect of antimalarial drugs on a preferential production of male or female gametocytes has not been reported. By comparison, there is increasing evidence that antimalarial drugs may influence sex ratio through preferential clearance of gametocytes of either sex. *In vitro* drug tests suggest that male gametocytes are more sensitive to a range of antimalarial drugs compared to female gametocytes, with impaired male gametocyte fitness (reduction in exflagellation) at drug concentrations that do not affect female gametocyte fitness (as measured by activation into **gametes**) [20]. Antifolates, for example, may disproportionately affect males by inhibiting the folate-mediated pyrimidine synthesis required for DNA replication during exflagellation [20]. Although a sex-specific effect of antimalarial drugs has been proposed as an explanation for the early sterilizing effect of transmission-blocking antimalarials [74], gametocyte sex ratio assessments are not commonly incorporated in antimalarial drug efficacy clinical trials. Despite initial reports of a reduced duration of male gametocyte carriage [75], and a relative decrease in the proportion of male gametocytes [76] following artemisinin-combination treatment (ACT), no obvious pattern of a sex-specific effect of ACT emerges when all available data are examined (Figure 2). On the other hand, most available (microscopy-based) data suggest that treatment with non-ACTs (such as sulphadoxine-pyrimethamine, amodiaquine and chloroquine) results in a male-biased sex ratio shortly after treatment 77, 78, 79 (Figure 2). Whilst a single trial with molecular gametocyte sexing tools did not confirm this effect for sulphadoxine-pyrimethamine plus amodiaquine [62], it is intriguing to consider if this explains earlier observations on enhanced infectivity early after treatment with non-ACTs [80]. Recent evidence suggests that adding a single dose of the gametocytocidal drug primaquine to ACT is associated with male-biased sex ratios in the first 14 days after treatment, followed by a normalization to a female-biased ratio 34, 62 that may be a consequence of gametocyte release, ongoing gametocyte production, or a longer circulation time of female gametocytes. The most pronounced sex-specific effect may be exerted by the gametocytocidal drug methylene blue that appears to preferentially clear male gametocytes 62, 81, 82.

One important outstanding question is whether transmission potential is predictable after treatment based on gametocyte density and sex ratio, or whether **mosquito feeding assays** remain essential to predict transmission potential after treatment 74, 83. The strong association between gametocyte density and mosquito infection rates that is observed before treatment 23, 84 may be retained 62, 85 or lost after treatment [84]. If the quantification of gametocyte sex ratio allows mosquito infection rates to be predicted with acceptable precision, this may obviate the need for mosquito feeding assays in antimalarial drug trials. Another scenario is that drugs may sterilize gametocytes, either of both sexes, or preferentially affecting one sex, without immediately affecting their circulation time. Under this scenario mosquito transmission assays will continue to be essential to estimate the effects of antimalarial drugs on transmission potential.

## Concluding Remarks

The transmission of *P. falciparum* to mosquitoes represents a developmental bottleneck in terms of parasite numbers; at the same time it is a very efficient process that may even increase in efficiency once transmission intensity declines [86]. For successful fertilization in the mosquito midgut, sufficient numbers of male and female gametocytes need to be generated during infections. Quantifying both male and female gametocytes therefore plausibly allows a better prediction of infectivity than the total gametocyte biomass or simple measurement of (the more abundant) female gametocytes [23]. Quantifying the densities of male and female gametocytes may also assist in the evaluation of the transmission-blocking properties of antimalarial drugs but at present cannot replace mosquito feeding assays to provide definitive evidence of post-treatment transmission potential (see Outstanding Questions). Changes in total parasite density or gametocyte density by antimalarial drugs or other environmental factors may promote malaria parasites to adjust their investment in transmission stages; this may be by increasing commitment to gametocytes or by increasing the proportion of male gametocytes to maximize transmission success [23]. Whilst of clear value in this respect, previously published assessments of gametocyte sex ratio are affected by the limited sensitivity of microscopy for gametocyte detection and sexing. With the recent development of molecular tools to enumerate male and female gametocytes, presumed environmental stimuli for sex ratio adjustment should be reconsidered. For such studies to provide reliable estimates, it is essential to acknowledge the presence of low levels of male and female gametocyte transcripts in both sexes and in asexual parasites, which necessitates a careful interpretation of gametocyte prevalence estimates in samples from patients with high total parasite burden and a careful interpretation of extreme sex ratios.

Outstanding Questions What are the sensing mechanisms and stimuli that allow malaria parasites to adjust their sex ratio?

Do male and female gametocytes differ in longevity or the duration of infectivity?

What range of gametocyte sex ratio allows transmission under different conditions?

Do different clones within the same infection vary in gametocyte sex ratio, and how does this affect their transmission?

Do antimalarial drugs prevent transmission by means of gametocyte sterilization rather than gametocyte clearance?

Can robust quantification of male and female gametocytes replace mosquito feeding assays?

## Glossary

**Controlled human malaria infection (CHMI)**: deliberate infection of a healthy noninfected human volunteer with malaria under highly standardized conditions. Infection happens either by intravenous inoculation of *Plasmodium*-infected erythrocytes or through the bite of sporozoite-infected mosquitoes. Upon reaching blood-stage infection, the volunteer receives treatment. In CHMI models aiming to study gametocyte development, treatment aims to eliminate asexual parasite replication while allowing gametocytes to develop and mature.
**Gametes**: sexually dimorphic parasite forms that develop from gametocytes activating in the mosquito gut. Female gametocytes give rise to a single female gamete (macrogamete), male gametocytes give rise to up to eight motile microgametes; each female gamete may be fertilized by a male microgamete.
**Gametocyte**: the sexual stage malaria parasite capable of reproduction in the mosquito. Female and male gametocytes circulate in the human peripheral blood, where they may be ingested by blood-feeding *Anopheles* mosquitoes and continue sexual development (see Gametes).
**Mosquito feeding assay**: an assay for determining the infectiousness of *Plasmodium* gametocytes to *Anopheles* mosquitoes. Mosquito feeding assay may refer to an assay in which mosquitoes are allowed to feed on the patient’s blood (skin feeding or direct membrane feeding assay after venous blood was drawn) or on cultured gametocytes (standard membrane feeding assay).
**Proportion of male gametocytes**: the proportion or percentage of all gametocytes that is male $\left(\frac{\text{male}\;\text{gametocytes}}{\text{male}+\text{female}\;\text{gametocytes}}\right)$.
**Proteomics**: studies that capture the entirety of proteins of a cell or organism, often at a certain life stage. Proteomics is usually based on mass spectrometry.
**Schizogony/erythrocytic schizogony**: creation of, on average, 16–32 merozoites in an infected red blood cell during the schizont stage of asexual replication. Schizogony is a rapid process in *P. falciparum* (∼48 h), and should not be confused with liver-stage (exoerythrocytic) schizogony in hepatocytes, which takes 6–7 days and produces thousands of merozoites.
**Sex determination**: used to describe the ‘decision’ to become either a male or female gametocyte, plausibly determined at the same time when sexual commitment happens.
**Sex ratio**: the ratio of male to female gametocytes $\left(\frac{\text{male}\;\text{gametocytes}}{\text{female}\;\text{gametocytes}}\right)$. This term is used inconsistently in the literature, referring to either sex, dependent on context. In the current review, we instead use the proportion of male gametocytes.
**Sexual commitment**: the route to the differentiation into a gametocyte, thereby leaving the asexual replication cycle. The exact timing of commitment is not entirely understood, but all merozoites derived from one committed schizont will develop into gametocytes of one sex.
**Transcriptomics**: studies that capture the entirety of transcripts of a cell or organism, often at a certain life stage. Transcriptomics is usually based on microarrays or RNA sequencing.
**Translational repression**: a phenomenon in which mRNA transcripts are not (yet) translated into proteins but accumulated and protected from degradation. Stored transcripts frequently support cellular processes immediately after fertilization when translation is needed but transcription is low or absent.
